# Grave thoraco-intestinal complication secondary to an undetected traumatic rupture of the diaphragm: a case report

**DOI:** 10.1186/s40001-021-00488-9

**Published:** 2021-02-08

**Authors:** Morris Beshay, Martin Krüger, Kashika Singh, Rainer Borgstedt, Tahar Benhidjeb, Edwin Bölke, Thomas Vordemvenne, Jan Schulte am Esch

**Affiliations:** 1 Department of Thoracic Surgery, University Hospital OWL of the University Bielefeld, campus Bielefeld-Bethel, Bielefeld, Germany; 2 Department of Internal Medicine and Gastroenterology, University Hospital OWL of the University Bielefeld, campus Bielefeld-Bethel, Bielefeld, Germany; 3 Department of General and Visceral Surgery, University Hospital OWL of the University Bielefeld, campus Bielefeld-Bethel, Bielefeld, Germany; 4 Department of Anesthesiology, Intensive Care, Emergency-, Transfusion- and Pain-Medicine, University Hospital OWL of the University Bielefeld, campus Bielefeld-Bethel, Bielefeld, Germany; 5 Medical Faculty, Department of Radiation Oncology, Heinrich Heine University, Duesseldorf, Germany; 6 Department of Trauma Surgery and Orthopedics, University Hospital OWL of the University Bielefeld, campus Bielefeld-Bethel, Bielefeld, Germany; 7 Department of General and Visceral Surgery, Evangelisches Klinikum Bethel, Schildescher Str. 99, 33611 Bielefeld, Germany

**Keywords:** Bowel perforation, Pneumothorax, Herniation, Diaphragmatic rupture, Pleural empyema, Case report

## Abstract

**Background:**

Diaphragmatic lesions as a result of blunt or penetrating trauma are challenging to detect in the initial trauma setting. This is especially true when diaphragmatic trauma is part of a polytrauma. Complications of undetected diaphragmatic defects with incarcerating bowel are rare, but as in our patient can be serious.

**Case presentation:**

A 57-year-old female presented to the Emergency Room of our Hospital in a critical condition with 3 days of increasing abdominal pain. The initial clinical examination showed peritonism with tinkling peristaltic bowel sounds of mechanical obstruction. A thoraco-abdominal CT scan demonstrated colon prolapsed through the left diaphragmatic center with a large sero-pneumothorax under tension. As the patient was hemodynamically increasingly unstable with developing septic shock, an emergency laparotomy was performed. After retraction of the left colon, which had herniated through a defect of the tendinous center of the left diaphragm and was perforated due to transmural ischemia, large amounts of feces and gas discharged from the left thorax. A left hemicolectomy resulting in a Hartmann-type procedure was performed. A fully established pleural empyema required meticulous debridement and lavage conducted via the 7–10 cm in diameter phrenic opening followed by a diaphragmatic defect reconstruction. Due to pneumonia and recurring pleural empyema redo-debridement of the left pleural space via thoracotomy were required. The patient was discharged on day 56. A thorough history of possible trauma revealed a bicycle-fall trauma 7 months prior to this hospitalization with a surgically stabilized fracture of the left femur and conservatively treated fractures of ribs 3–9 on the left side.

**Conclusion:**

This is the first report on a primarily established empyema at the time of first surgical intervention for feco-pneumothorax secondary to delayed diagnosed diaphragmatic rupture following abdomino-thoracic blunt trauma with colic perforation into the pleural space, requiring repetitive surgical debridement in order to control local and systemic sepsis. Thorough investigation should always be undertaken in cases of blunt abdominal and thoracic trauma to exclude diaphragmatic injury in order to avoid post-traumatic complications.

## Background

The incidence of traumatic diaphragmatic rupture in trauma patients is about 0.5% [1]. With 80% of those cases, the left side is predominantly involved, with two-thirds of the cases subsequent to penetrating injuries and the remaining as a result of blunt trauma [1, 2]. Up to 3% of abdominal traumas include diaphragmatic tears that bear a diagnostic challenge as they can only reliably be ruled out with direct visualization via laparo- or thoracoscopy and remain primarily undetected in 50% of cases [3–5]. Although prolapse of intestinal content at the site of the traumatic diaphragmatic hernia is a known presentation, severe forms are rare.

## Case presentation

**Fig. 1:**
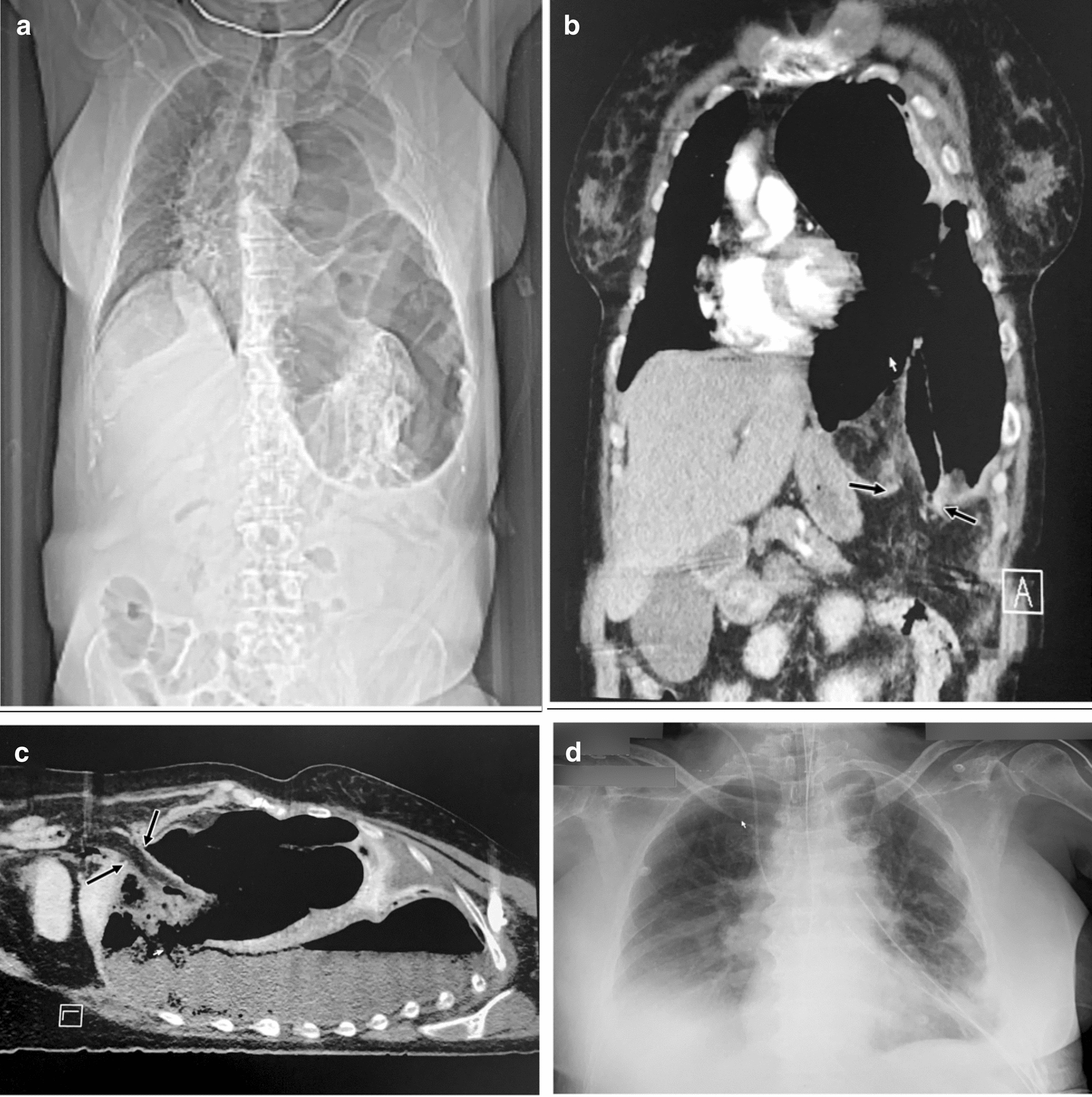
Radiological imaging on day of admission. Thoraco-abdominal CT scan with localizer of carried out CT scan (**a**) and representative CT scan images (**b**, **c**): enterothorax with splenic flexure prolapse through an old undetected traumatic rupture of the left diaphragmatic center, consecutive colic incarceration (**b** + **c**; arrows) with ischemia, rupture of the colon into the left pleural cavity with feco-pneumothorax under tension, cardiac depression due to mediastinal shift to the right and sepsis secondary to left pleural empyema (**c**), inversed diaphragm and midline shift to the right (**a** + **b**). Post-surgical chest X-ray examination (**d**): re-expanded left lung and reversal of the mediastinal shift with reconstructed diaphragm

**Fig. 2:**
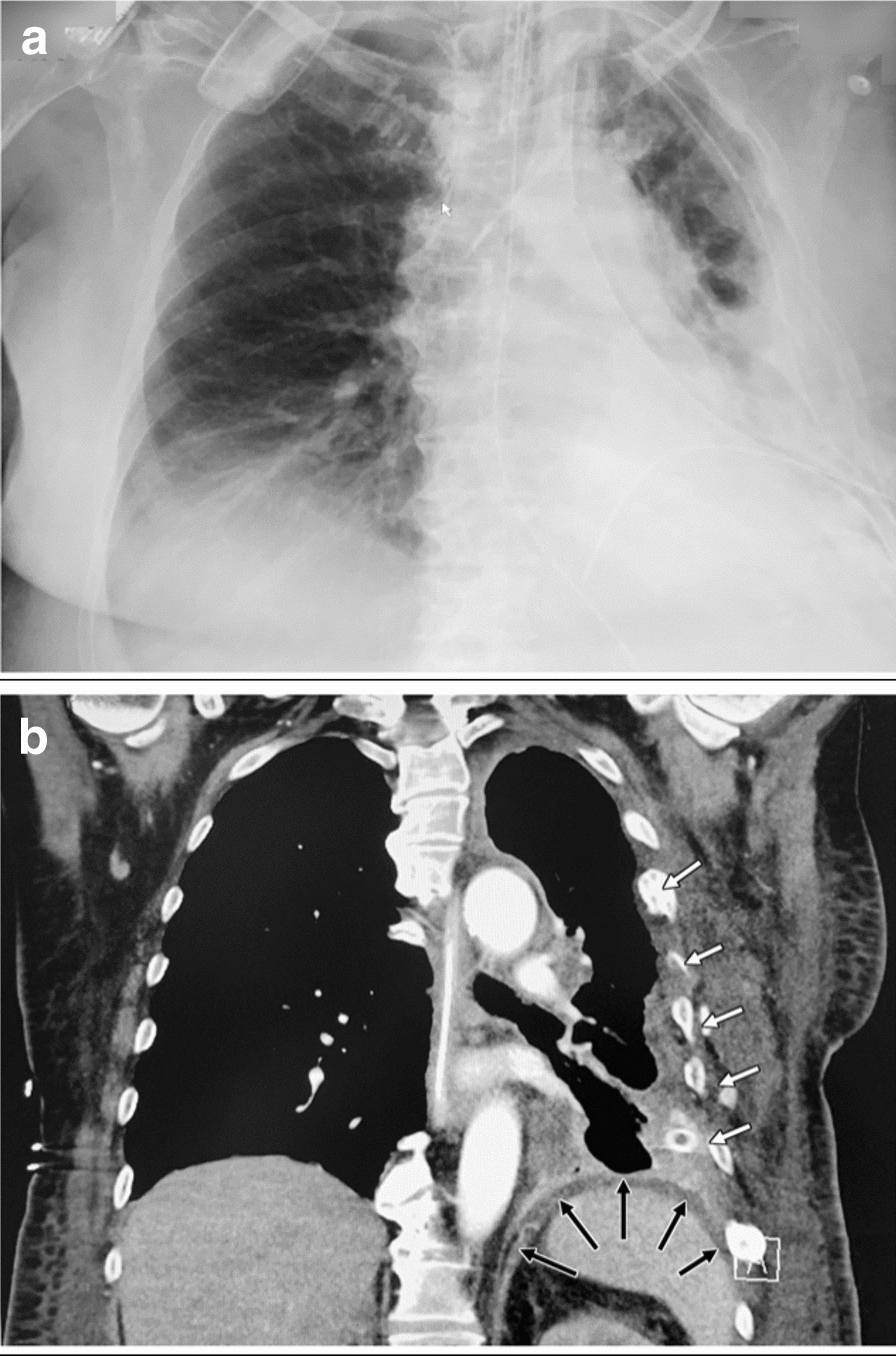
Radiological imaging on day 20. Chest X-ray examination (**a**) and thoraco-abdominal CT scan (**b**): reaccumulation of the left pleural empyema. The diaphragm stayed intact (black arrows) over the whole clinical course subsequent to reconstruction during initial surgery on day 1. White arrows indicate remnants of the healed rib fractures as a consequence of the bicycle trauma 7 months prior to admission for feco-pneumothorax

A 57-year-old female presented to the Emergency Room in a critical condition, severe abdominal pain (NRS 8/10), diarrhea, nausea without vomiting or dyspnea. Abdominal pain was present for 3 days, initially intermittent and colicky changing to a constant and sharp quality with increasing intensity. She reported no co-morbidity and a hysterectomy 29 years ago. The initial clinical examination showed the following findings: the patient was hemodynamically increasingly unstable with marbled skin color. Peritonism was present with tinkling peristaltic bowel sounds of mechanical obstruction. The patient was in a septic shock with severe lactate acidosis (9.5 mmol/l; pH 7.279), a base excess of -16.0 mmol/L and an O_2_-saturation of 84% prior to receiving high flow oxygen. Further laboratory testing revealed elevated creatinine (3.0 mg/dl), increased C-reactive protein (407.9 mg/l, ref.: < 5), interleukin-6 of 14,864.0 pg/ml and a procalcitonin of 37.1 ng/ml. E. coli was subsequently detected in blood cultures. CT scan revealed prolapsed colon through the left diaphragmatic center with sero-pneumothorax under tension (Fig. 1a–c). While preparing for emergency laparotomy, the patient had a cardiac arrest requiring CPR for 5 min. An immediate laparotomy and retraction of the splenic flexure and left colon, which herniated through a defect of the tendinous center of the left diaphragm accompanied by a perforation due to transmural ischemia, was performed. Two liters of feces and a large amount of gas were evacuated from the left thorax. A left hemicolectomy with an end transverse colostomy and sigmoid closure resulting in a Hartmann-type procedure was performed. Rigorous pleural lavage via the traumatic tear of 7–10 cm in diameter unmasked an empyema with a pleural membranous thickening of up to 8 mm. Meticulous debridement of the lower lobe and caudal parts of the upper lobe trans-diaphragmatically was conducted via the diaphragmatic opening was followed by a defect reconstruction with a woven running suture in two rows. Subsequently, the reconstructed diaphragm stayed intact for the total clinical course as revealed by chest X-ray examination on postoperative day 3 (Fig. 2). Due to pneumonia and recurring pleural empyema in the course of a prolonged ICU-stay, repetitive bronchoscopies and two redo debridements of the left pleural cavity via open thoracotomy were required. Weaning of ventilation was supported by temporary tracheostomy. A wound infection of the laparotomy wound was managed with vacuum dressing followed by secondary closure. The patient was discharged on day 56. A thorough history of possible trauma revealed a bicycle-fall trauma 7 months prior to this hospitalization with a fracture of the left femur, stabilized by internal fixation, and conservatively treated fractures of ribs 3–9 on the left side.

## Discussion and conclusion

Feco-pneumothorax due to perforation of diaphragmatically herniated colon to the pleural cavity is a rare entity, and frequently associated with a tension pneumothorax unmasking the diaphragmatic lesion weeks to decades after the initially undetected injury [6, 7]. Nine of a total of 16 cases previously reported were secondary to perforating chest injuries [3, 6, 8–14], whereas the remaining seven cases had a history of blunt abdominal and/or thoracic injury as happened to our patient [4, 7, 15–19]. Empyema is a complication that can develop in the course of these patients, requiring secondary drainage or interventional debridement and is still associated with a mortality of 25–66% [3, 4]. Our patient is the second case of a primarily established empyema at the time of first surgical intervention out of a total of eight reported cases with feco-pneumothorax secondary to delayed diagnosed diaphragmatic rupture following abdomino-thoracic blunt trauma with colic perforations to the pleural space, the first reported in 1981 [7]. Further, it is the first case in that cohort requiring repetitive surgical debridement in order to control local and systemic sepsis.

Our patient had a rare and life-threatening variant of a delayed intestinal complication following an initially undetected traumatic diaphragmatic rupture. The diagnosis of traumatic diaphragmatic injury is usually challenging, even if a secondary or even third diagnostic survey according to ATLS principles is obtained. Therefore, thorough investigation should always be undertaken in cases of blunt abdominal and thoracic trauma to exclude diaphragmatic injury. In order to avoid overlooking such traumatic complication, both radiologists and surgeons should be familiar with the variety of changes potentially indicating diaphragm rupture, such as diaphragm discontinuity, collar sign, herniation, gas and fluid shadowing in the thoracic space, atelectasis in the lower lobes and dependent viscera sign like elevated abdominal organs [20, 21]. Further signs indicative for advanced or complicated diaphragmatic ruptures on pulmonary radiographs are pleural effusion, pneumothorax, hydro-pneumothorax and mediastinal shift, as observed for our patient.

The defect was a grade III of V according to “The Organ Injury Scaling Committee of the American Association for the Surgery of Trauma” as in the range of 2–10 cm length [22]. We selected a double-layer running suture, as advocated also by others for these sized defects of the ruptured diaphragm [1]. Although no larger or randomized trials are available on diaphragmatic reconstruction subsequent to trauma, recommendations range from monofilament non-absorbable or absorbable interrupted sutures for small defects of up to 6 cm to interrupted figure-of-eight or interlocking mattress sutures for larger defects. Rarely, there may be an indication for a prosthetic mesh for the larger defects [1]. The access can be abdominal, combined abdomino-thoracic or thoracic only, depending on the individual strategy and pattern of injuries. In our case, abdominal access was justified for the primary intervention.

Our strategy for treating postoperative recurrent empyema was based on three main principles: (1) broad spectrum antibiotics as early as possible; (2) complete surgical debridement and lavage with or without vacuum application, and (3) early mobilization and bronchial lavage. In accordance to the literature, we followed this very successful concept for the effective management of this patient’s postoperative empyema [23].

The severity of this patient’s condition illustrates the vital importance of early diagnosis of diaphragmatic ruptures following thoraco-abdominal trauma. Further, the importance of this report is to demonstrate that an inter-disciplinary treatment strategy involving diverse medical and surgical expertise is required, including rigorous ongoing pleural and bronchial management in order to obtain the optimum outcome.

## Data Availability

The data generated during and/or analyzed during the current case report are available from the corresponding author on reasonable request.The data generated during and/or analyzed during the current case report are available from the corresponding author on reasonable request.
